# Modeling the Role of Negative Cooperativity in Metabolic Regulation and Homeostasis

**DOI:** 10.1371/journal.pone.0048920

**Published:** 2012-11-09

**Authors:** Eliot C. Bush, Anne E. Clark, Chris M. DeBoever, Lillian E. Haynes, Sidra Hussain, Singer Ma, Matthew M. McDermott, Adam M. Novak, John S. Wentworth

**Affiliations:** Department of Biology, Harvey Mudd College, Claremont, California, United States of America; Semmelweis University, Hungary

## Abstract

A significant proportion of enzymes display cooperativity in binding ligand molecules, and such effects have an important impact on metabolic regulation. This is easiest to understand in the case of positive cooperativity. Sharp responses to changes in metabolite concentrations can allow organisms to better respond to environmental changes and maintain metabolic homeostasis. However, despite the fact that negative cooperativity is almost as common as positive, it has been harder to imagine what advantages it provides. Here we use computational models to explore the utility of negative cooperativity in one particular context: that of an inhibitor binding to an enzyme. We identify several factors which may contribute, and show that acting together they can make negative cooperativity advantageous.

## Introduction

Most enzymes operate in multi-subunit complexes, and a significant proportion, perhaps 25% or more, display cooperativity in binding ligand molecules [1]–[4]. This pheonomenon plays an important role in a number of cellular processes, among them metabolic regulation [5], [6].

**Figure 1:**
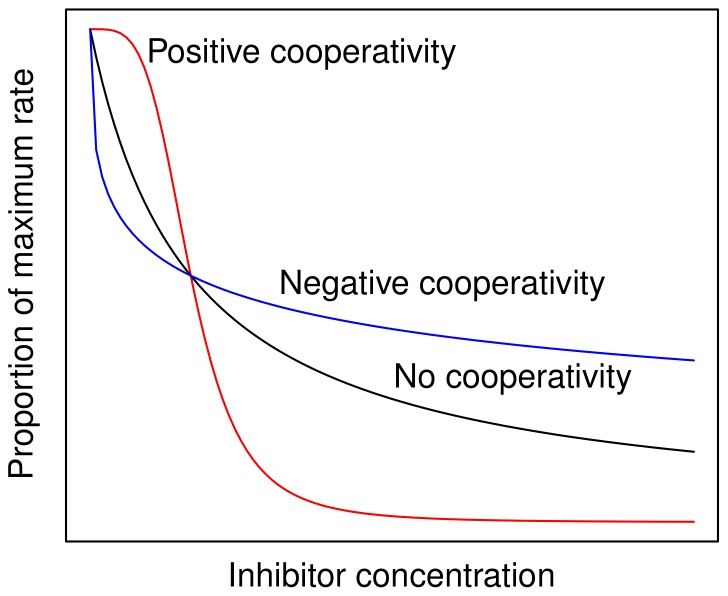
Positive and negative cooperativity in inhibitor binding an enzyme.

Here we consider the impact of cooperativity on metabolic regulation in the case of inhibitor binding to an enzyme. In this situation binding at one subunit can influence the affinity of other subunits for the same inhibitor, thereby affecting how the rate of catalysis responds to inhibitor concentration. In fig. 1 the red curve shows positive cooperativity, where inhibitor binding to one subunit causes additional subunits to have a higher affinity for the inhibitor. The blue curve shows negative cooperativity, where inhibitor binding causes other subunits to have a lower affinity.

End-product inhibition provides a natural context for examining cooperativity in metabolic regulation, and a number of studies have touched on its impact in this case [7]–[11]. One important conclusion has been that positive cooperativity can be advantageous because it allows a system to respond to environmental changes while minimizing the deviation of some key metabolite from its ideal concentration [10].

The value of negative cooperativity in metabolic regulation has received less attention. However this represents an important question because negative cooperativity is almost as common as positive among enzymes in nature [12], [13]. One hypothesis for the use of negative cooperativity suggests it is associated with branch points in metabolic networks [13]. Below we use computational models to explore this possibility and others, and identify several factors which can contribute to making negative cooperativity advantageous.

## Results and Discussion

### A simple example metabolic network

**Figure 2:**
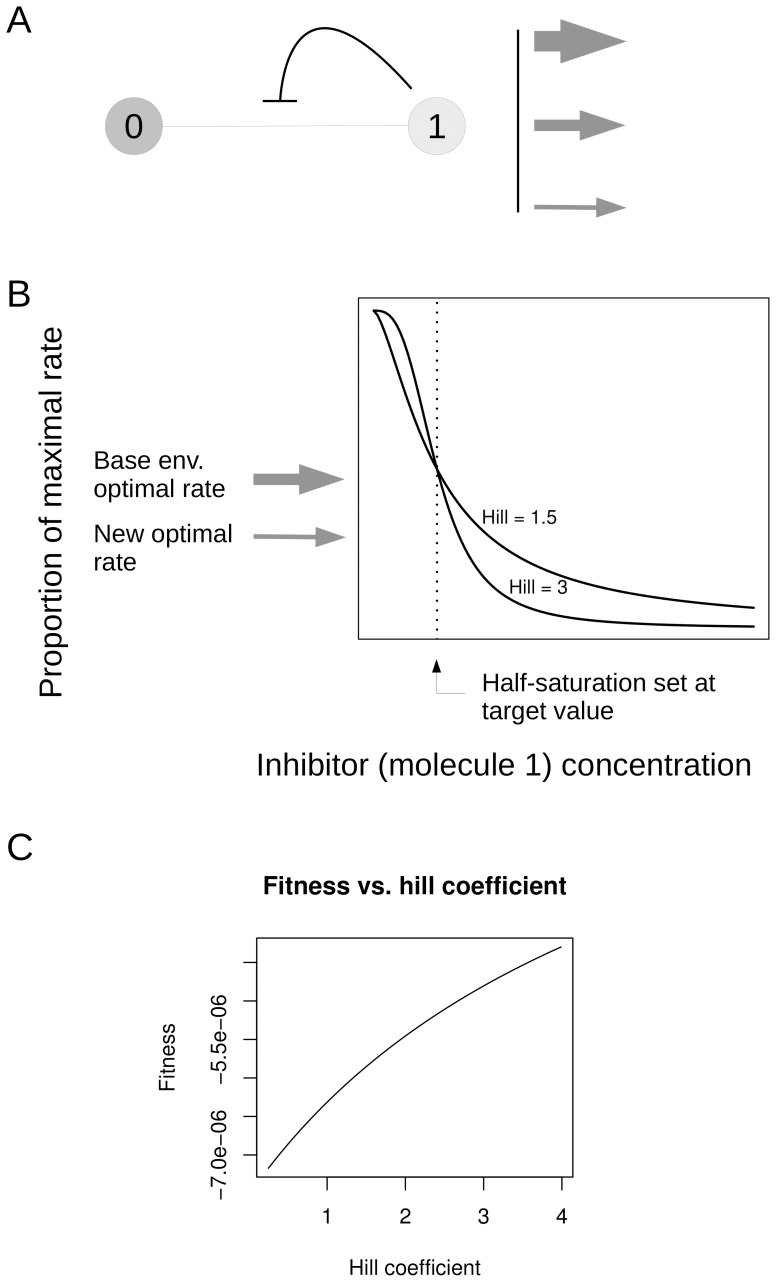
A simple example network with regulation. A. The network consists of two molecules connected by one reaction. The reaction is inhibited by the product molecule 1. There are fixed rate outflow reactions from molecule 1. These take on different levels in different environments, as illustrated by the arrows. B. Plot of the activity of the enzyme vs. concentration of the inhibitor (molecule 1). The two curves have half-saturation at molecule 1's target value, and illustrate the shape with two different Hill coefficients. C. A plot showing how fitness varies with Hill coefficient for this network, given that the half-saturation is set to molecule 1's target. Fitness (see text for definition) reflects the deviation of molecule 1 from its target and thus is in units of concentration.

Consider the metabolic network in fig. 2 A, a simplified version of that discussed in Hofmeyr and Cornish-Bowden 1991 [10]. The network consists of two molecules connected by a reversible Michaelis-Menten reaction. Molecule 0 is the precursor whose concentration is held constant by reactions outside the network. Molecule 1 is the product molecule which is subject to a constant rate outflow. This outflow varies in different environments. In our scheme there are three environments defined by three possible levels of outflow (arrows in fig. 2 A). We are interested in how metabolic homeostasis can be maintained in these environments.

We explore this computationally using simple kinetic models of enzymatic reactions. In the network in fig. 2 A, we first define a target concentration for molecule 1. Effective homeostasis will involve keeping molecule 1 as close as possible to this concentration. We establish the target by treating the environment with the middle level of outflow as a sort of baseline environment. We determine the steady state concentration of molecule 1 for this baseline environment given a particular set of parameters (enzyme amount, molecule energies and so on). This steady state value becomes the target concentration which the system is trying to maintain. We then examine what happens when the rate of outflow is changed. Starting the system at the target concentration, we see how molecule 1 concentration deviates when outflow is raised or lowered.

In the absence of regulation a new steady state will be reached via mass action. In the case of a lowered outflow, the concentration of molecule 1 will begin to build up. The rate of the back reaction (1
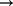
0) increases relative to the forward reaction. This continues until the net flow though the reaction has been reduced to the same level as the new reduced outflow, and a new steady state is reached.

Regulation can allow the system to achieve a new steady state while minimizing the deviation of molecule concentrations from their target values. Regulation in our system is achieved through the action of one or more non-competitive inhibitors on an enzyme. We model inhibitor binding with the Hill equation which has been shown to provide a good fit to ligand binding data in a wide variety of situations [14], including situations with negative cooperativity [15]–[17]. We regard this equation as being empirical and do not base it on an explicit physical model of binding. In our set up an enzyme with an inhibitor has two parameters of interest: the Hill coefficient and the half-saturation point for inhibitor binding. The Hill coefficient controls the sharpness of the enzyme's response to inhibitor, and the half-saturation point determines the inhibitor concentration at which enzyme reaches half its maximum rate.

A regulated solution to the network in fig. 2 A involves end-product inhibition of the enzyme by molecule 1 which we imagine binding at an allosteric site. The system must respond to deviations in outflow which go in either direction. We can achieve this by doubling the amount of enzyme relative to what was used to establish the target values, and setting the half-saturation point for inhibitor binding to the target value for molecule 1. Thus when molecule 1 is at its target value, half the enzyme sites will be bound. With twice as much enzyme, but half of it inhibited, the activity of the enzyme will be the same as it was when the target values were established, and deviations of molecule 1 concentration in either direction will produce the desired regulatory effect. Under these circumstances a high Hill coefficient (i.e. sharp inhibitor binding curve) leads to a new steady state minimizing the deviation of molecule 1 from its target value. The curves in fig. 2 B illustrate this. Molecule 1's concentration starts out at the target value shown by the dashed line. When the outflow is reduced, molecule 1's concentration starts to increase. As this happens we slide down the inhibition curve until the flow through the reaction matches the new outflow and we are at steady state. As can be seen from the figure, a steeper curve allows us to arrive at steady state while minimizing molecule 1's deviation from target. In the case of increased outflow, a steeper curve is also beneficial, by a similar argument.

A systematic examination of the relationship between metabolic homeostasis and Hill coefficient in this network is consistent with our expectation. For a given Hill value we can calculate the deviation of molecule 1 from its target in all environments. We define fitness as the negative of the average of the absolute values of these deviations. The larger the fitness, the better the homeostasis. We examined fitness for Hill coefficients from 0.25 to 3.98 for this simple network, and the results are shown in Fig. 2 C. As expected, fitness is highest with the maximum possible Hill.

### The Hill coefficient and branching pathways

**Figure 3:**
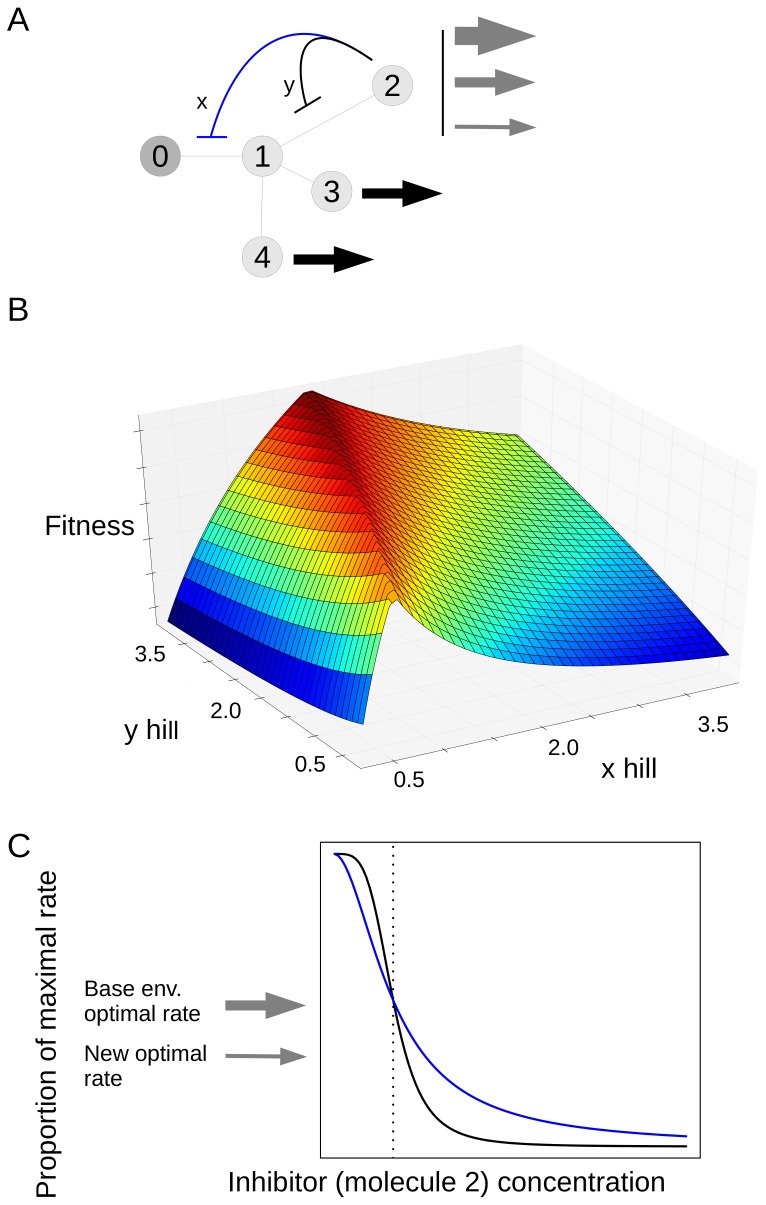
A branching network where the best fitness is not given by the maximum Hill coefficient. A. Illustration of the network. Molecules 3 and 4 have constant rate outflows which do not vary in different environments, shown with black arrows. B. A fitness landscape for this network. The x and y axes give Hill coefficient for inhibition of the x and y reactions. The z axis is fitness, which reflects the deviations of molecules from their targets and is in units of concentration (see text for definition). C. Plot of the activity of the enzyme vs. concentration of the inhibitor (molecule 2).

We next consider a variation on this situation where the maximum possible Hill does not give the fittest solution. Like our first example, the network shown in fig. 3 A begins with a precursor molecule 0 whose concentration is fixed by external factors. However in this case molecule 1 is not the product, but rather represents a branch point in the network. It has reactions with molecules 2, 3 and 4 which are themselves the products of the pathway. Each of these products is attached to a constant rate outflow reaction. The outflow through molecules 3 and 4 always has the same level, however the outflow through 2 can take on three different levels, corresponding to three different environments.

We are once again interested in finding a way to regulate this network to maintain metabolic homeostasis. We now have multiple molecules and we will consider homeostasis to involve minimizing the deviation of all of them from their targets. Thus fitness will be defined as follows. We determine the deviations of all molecules from their targets, considered across all environments. The negative of the average of the absolute values of these deviations is the fitness.

Fig. 3 A shows a regulatory scheme for this network. Molecule 2, which can be subject to different rates of outflow, acts as inhibitor to two reactions, x and y, a so-called nested pattern [9]. We examined the optimal inhibitor binding characteristics under this scheme using an evolutionary approach. A particular solution to this network consists of Hill coefficients and a half-saturations for inhibitor binding for each of reactions x and y. We evolved populations of solutions to this problem, subject to mutation and selection, over a period of generations (details in the Methods). For this network we performed 32 independent runs, all of which yielded essentially the same best solution in the final generation: the Hill and half-saturation values for all 32 best solutions were within 3.7% of each other. The half-saturation values were at the target value for molecule 2. The Hill coefficient for reaction y was 3.98, and for reaction x was 1.81 (the possible range of Hills was 0.25 and 3.98).

The relationship between Hill coefficients and fitness is shown by the fitness landscape in fig. 3 B. We set the half-saturation values to the target for molecule 2, and then systematically varied the Hill coefficient for reactions x and y over 374 values between 0.25 and 3.98. For each combination we calculated the fitness. In fig. 3 B, fitness is represented along the z axis, and Hill values for the two reactions along the x and y axes. The figure shows clearly that the highest fitness values occur when Hill for reaction y is at the maximum, but the Hill for reaction x is less than the maximum.

To understand why it is advantageous to have the reaction x Hill less than the maximum, consider the plot in fig. 3 C. We have the inhibitor after the branch in black, and the one before in blue. Like we did above, lets consider what happens when we're in an environment where the flow out of this top branch has dropped. For the inhibition in black, the maximum possible Hill coefficient will be best for the reasons found above. This will not be the case for the reaction before the branch. We also want to inhibit this reaction, since we'll be needing less material moving through it due to the drop in outflow on the tip. The key point is that the percentage change of activity on this pre-branch enzyme should be less than the percentage change after the branch. This is because the pre-branch enzyme also has material going into the other branches, and the outflow through these has not changed. So we want to change the activity of the pre branch enzyme by a smaller proportion. Given that they both have half-saturations set at the target, the only way to achieve this is to have a less steep curve.

We looked for this pheonomenon over a range of conditions, varying enzyme amount and the magnitude of the baseline outflow over an order of magnitude. We find that for a wide range of parameter values, the best Hill value is less than the maximum possible. The size of the effect is dependent on the magnitude of the net flow through reaction x relative to the magnitude of the back reaction through x. The effect is most robust if the flow is greater than or equal to about 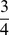 of the back reaction (in the base environment).

### The Hill coefficient and long pathways

**Figure 4:**
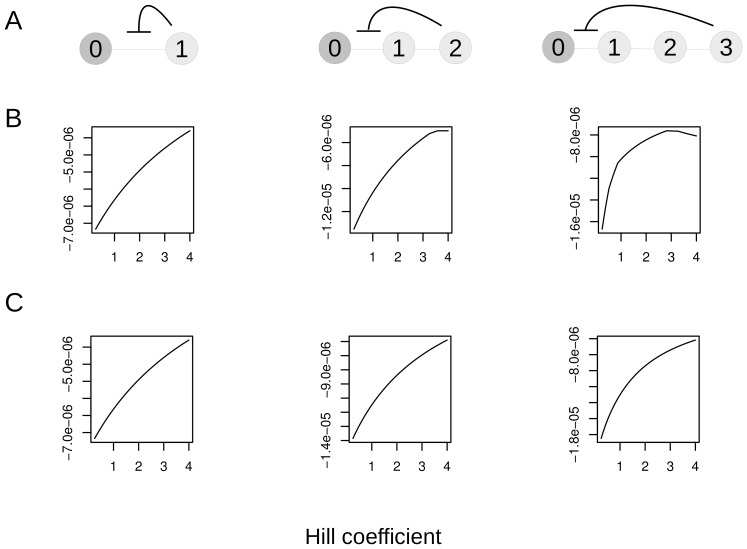
Several longer pathways where the best fitness is not given with the maximum Hill coefficient. A. Pathways with 2, 3 and 4 molecules. B. Plots of fitness vs. Hill coefficient of inhibitor binding given homeostasis on all molecules. C. Fitness vs. Hill coefficient given homeostasis acting only on the tip molecule. Fitness reflects the deviations of molecules from their targets and is in units of concentration (see text for definition).

Lets next consider what happens when we extend the simple pathway in fig. 2 to include one or two intermediate molecules. This is shown in fig. 4 A. As was the case in the last example, homeostasis here will involve minimizing the deviation of all molecules from their targets.

We again used an evolutionary approach to search for the optimal combination of Hill coefficients and half-saturations for these networks. For our example set of parameters (see Methods) for all three pathway lengths the best solutions involved half-saturations set to target values. Optimal Hill's for the 2, 3 and 4 molecule cases were 3.98, 3.59, 2.81 respectively. So the optimal Hill declines as the pathway becomes longer. We illustrate this in fig. 4 B, where we have calculated fitness for a range of Hill values for the three networks, setting half-saturation at target.

These longer networks are very similar to what was considered by Hofmeyr and Cornish-Bowden [10]. Those authors found that the higher the Hill coefficient the better. The difference between their results and ours lies in the definition of homeostasis. Hofmeyr and Cornish-Bowden were only considering homeostasis for the final product molecule, whereas we imagine homeostasis acting on all the molecules in the pathway, a situation which has been argued to be more realistic [11], [18]. Fig. 4 C shows fitness by Hill if we only have homeostasis acting on the tip molecule as in ref [10].

The reason for the drop in optimal Hill for longer pathways is that there is a delay in concentrations equilibrating in the long branch. Let us consider what happens in the 4 molecule network when we test with the lower outflow environment. Imagine that the Hill coefficient is at the maximum possible value. The inhibitor concentration (molecule 3 in this case) begins to increase. We slide down the inhibition curve, and at some point inhibition causes the rate of flow through the inhibited reaction to equal the new rate of outflow from the product molecule. But the system is not yet at steady state because the two internal reactions do not yet have the same rate as the new outflow. The first reaction can reach that level quickly through inhibition. But the other two will need to do so through mass action, which takes longer. Their equilibration will cause the concentration of molecule 3 to continue to increase, leading to overinhibition, and the system eventually settling in a steady state that undershoots the final target concentrations. In this situation, fitness can be optimized by having a less steep inhibition curve which does not constrict entry into the pathway as quickly.

We systematically explored parameter space for this system, to try to understand the conditions under which the optimal Hill coefficient drops when we go from two to three molecules in our pathway. Using the range of Hill coefficients used above (0.25–3.98), we found that roughly 20% of parameter combinations produced this effect. In another 50% of the cases the optimal Hill for both two and three was at the maximum of 3.98, but if we allowed ourselves to go to Hills above this value we found that the effect was still present (i.e optimal Hill for three was less than that for two).

Because this effect depends on the gradient of concentrations that is established in a pathway with flow going through it, the relative energies of the molecules in that pathway will impact it. We found that running our examples in energetically unfavorable pathways tends to make our effect stronger (i.e. less-than-maximum Hill coefficients are more useful in longer pathways), and running them in energetically favorable pathways tends to reduce the magnitude of the the effect.

### An example with negative cooperativity

**Figure 5:**
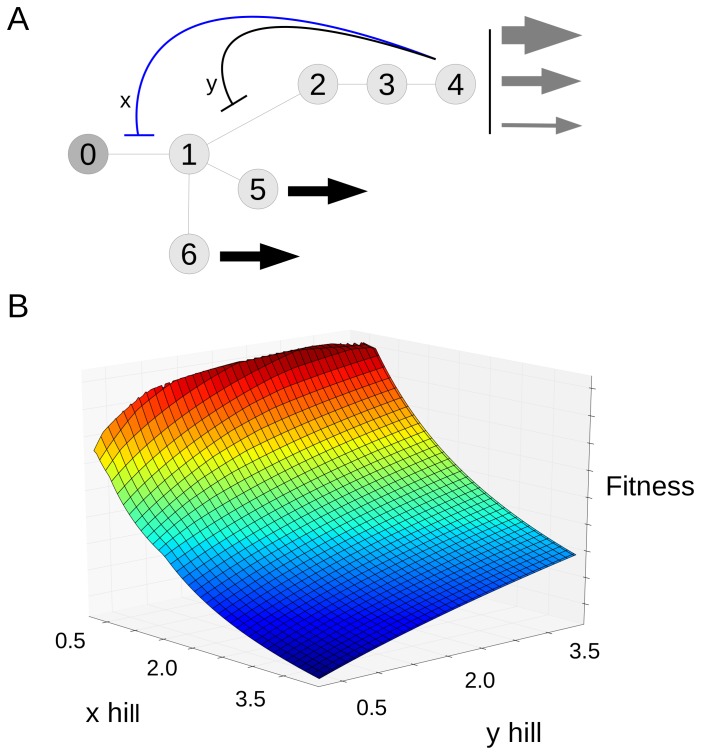
Example of a network with negative cooperativity of inhibitor binding (A). B. A fitness landscape for this network. The x and y axes give Hill coefficient for inhibition of the x and y reactions. The z axis is fitness, which reflects the deviations of molecules from their targets and is in units of concentration (see text for definition).

Our goal is to identify advantages of negative cooperativity in metabolic systems. So far we have described two phenomena which result in less-than-maximum Hill values, but not negative cooperativity. Using relatively extreme parameter values (e.g. a branching network with 12 branches) we found that we could get negative cooperativity to be advantageous. However we would like to find less extreme cases where this is so. We next discuss a small network which exhibits negative cooperativity in inhibitor binding (fig. 5 A). This network is similar to the branching network discussed above, except the branch with multiple levels of outflow has been extended by two additional reactions. This network thus combines the two properties discussed above, branching and having a pathway with one or more intermediates.

We again used an evolutionary approach to explore the optimal solutions for this network. In this case, we decided to not only vary the Hill and half-saturation on reactions x and y, but to also allow the identity of their inhibitors to vary. This made the parameter space we explored larger, but also gives us more confidence that we are finding the global optimum solution for the network.

We did 32 independent runs, and the best solution in each of these followed the same general pattern. They all involved molecule 4 inhibiting both reaction x and y, half-saturations for both reactions set around molecule 4's target value, and negative cooperativity (Hill
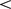
1) in inhibitor binding for reaction x. The ranges of parameter values from the best organisms in these 32 runs are shown in table 1.

**Table 1:** Evolved solutions for the network in fig. 5 A.

reaction x Hill	0.34–0.51
reaction y Hill	1.59–3.32
reaction x half-saturation	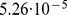 – 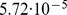
reaction y half-saturation	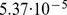 – 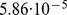

Range of Hills and half-saturations for best organisms from 32 runs on the network in fig. 5 A. Note that the target for molecule 4 is 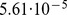 M.

This network provides an illustration of how negative cooperativity can arise in a metabolic network. It combines the two phenomena we have discussed above. First it is a branching network with nested end-product inhibition. Second the pathway producing this end-product has several intermediates. Our results show that acting together, these two phenomena are capable of producing negative cooperativity, at least under certain sets of parameters.

Before concluding, we would like to address one objection which could be made to our approach. We have used the Hill equation to model inhibitor binding on the basis that it provides a good empirical fit to ligand binding data [14]. One might object that it would be preferable to use an equation which is based on an explicit physical model. It is attractive to use equations based on explicit models, however the great disadvantage here is their complexity. To have cooperativity values over a sufficiently large range, we would need to use models with more than two interacting subunits. The resulting equations would have huge numbers of terms. Given that our chief interest is in understanding metabolic regulation at the network level, we feel it is justified, and indeed strongly preferable to use simpler empirically based equations to represent inhibitor binding. The Hill equation does this, fitting binding data well, including in negatively cooperative systems [15]–[17]. We note that other workers have made similar choices [19].

## Conclusions

We have suggested two factors to explain the evolution of negative cooperativity in metabolic regulation. First, in branching networks with nested end-product inhibition, it is advantageous for the inhibitor to bind the enzyme before the branch with a smaller Hill coefficient than it binds the one after. This is because the branch point enzyme needs to be modulated less strongly than enzymes after the branch. If both are inhibited by the same end product, then it is optimal for the branch point enzyme to have a less sharp response to inhibitor. The second factor occurs in linear, unbranching pathways with end-product inhibition. It is due to a difference in the time scale between inhibition on the one hand, and mass action on the other. An important condition for this second factor is that metabolic homeostasis be acting on intermediates as well as products of the pathway, a situation which is likely to be common in nature [11], [18].

We have used simple models to demonstrate that these two factors occur over a range of parameter values, and that in combination they can lead to negative cooperativity. A natural extension would be identify pathways which are known to exhibit end product inhibition with negative cooperativity in nature [20], [21], and model them in greater detail.

Our theoretical results are a step toward understanding the role of negative cooperativity in metabolic regulation. Beyond its intrinsic interest, this has the potential to aid biotechnology as humans seek to design and modify metabolic pathways for our own purposes.

## Methods

Our numerical simulations of chemical reactions were written in python using the scipy package's odeint function. Our code can be downloaded from our website at http://proconsul.bio.hmc.edu/lp/.

We used a two-step reversible Michaelis-Menten mechanism for our reactions:
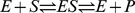
Our simple model of inhibition allows for one or more non-competitive and cooperatively binding inhibitors to bind an enzyme and inactivate it. We treat the process of inhibitor binding as being rapid compared to substrate binding, and use the Hill equation to determine the proportion of enzyme which is unbound and therefore active.

This gives us the following rate equations:

No inhibitor:
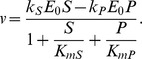
n independently acting non-competitive and cooperatively binding inhibitors:
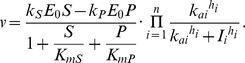



Here 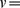 reaction rate; 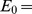 total enzyme concentration; 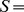 reactant concentration; 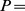 product concentration; 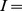 inhibitor concentration; 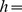 Hill coefficient of inhibitor binding; 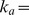 half-saturation point of inhibitor binding; 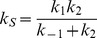; 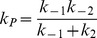; 
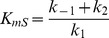
; and 
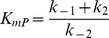
.

We obtained the various rate constants (e.g. 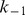 etc.) using the Arrhenius equation, with temperature 298 K, pre-exponential factor of 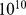, and the activation energies for binding and the main reaction at 10 and 30 kJ/mol respectively. All enzymes shared these parameters.

The network in fig. 2 A was constructed with the following parameters. The source molecule 0 had its concentration fixed at 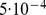 M, the enzymes had a concentration of 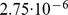 M, and the baseline rate of outflow from molecule 1 was 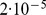 M/s. We varied the rate of outflow by a factor of 1.18 in the different environments, and all molecules had the same energy. The networks in fig. 4 A had the same parameters. And those in fig. 3 A and fig. 5 A differed only in having different concentrations of enzyme, 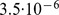 and 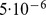 M respectively.

We systematically explored the space of parameters for several of our networks (fig. 3 A and fig. 4 A for the 2 and 3 molecule cases). For these runs we varied enzyme amount between 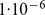 M and 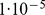 M using a step size of 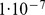 M. We varied the baseline rate of outflow between 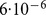 M/s and 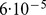 M/s with a step of 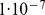 M/s. We varied the Hill coefficients between 0.25 and 3.98 with a step of 0.1. Half-saturations were set to the target values for the inhibitor, and other parameters were as above.

Our evolutionary simulations involved mutation and selection without recombination. The simulation for fig. 3 used a population size of 200 of which the 60 most fit were selected in every generation. 32 replications were each run out for 1000 generations. The simulations for fig. 4 also had 32 replications, but were run for 500 generations. With the simulations for fig. 5, because of the larger parameter space to explore, we found it useful to use significantly larger population sizes. These had a population size of 3200 of which 1600 were selected every generation. Again 32 replications were run for 1000 generations.

## References

[1] Hopkinson DA, Edwards YH, Harris H (1976) The distributions of subunit numbers and subunit sizes of enzymes: a study of the products of 100 human gene loci. Annals of human genetics 39: 383–392. 782337 10.1111/j.1469-1809.1976.tb00144.x

[2] Hill CM, Waight RD, Bardsley WG (1977) Does any enzyme follow the Michaelis-Menten equation? Molecular and Cellular Biochemistry 15: 173–178. 887080 10.1007/BF01734107

[3] Traut TW, Evans DR (1988) Enzymes of nucleotide metabolism: the significance of subunit size and polymer size for biological function and regulatory properties. Critical Reviews in Biochemistry and Molecular Biology 23: 121–169. 10.3109/10409238809088318 3048887

[4] Traut TW (1994) Dissociation of enzyme oligomers: a mechanism for allosteric regulation. Critical Reviews in Biochemistry and Molecular Biology 29: 125–163. 8026214 10.3109/10409239409086799

[5] Monod J, Changeux JP, Jacob F (1963) Allosteric proteins and cellular control systems. Journal of molecular biology 6: 306. 13936070 10.1016/s0022-2836(63)80091-1

[6] Koshland DE (1968) Regulatory control through conformation changes in proteins. Advances in Enzyme Regulation 6: 291–301. 5720338 10.1016/0065-2571(68)90018-6

[7] Savageau MA (1974) Optimal design of feedback control by inhibition. Journal of Molecular Evolution 4: 139–156. 4469274 10.1007/BF01732019

[8] Savageau MA (1975) Optimal design of feedback control by inhibition. Journal of Molecular Evolution 5: 199–222. 1159800 10.1007/BF01741242

[9] Savageau MA (1976) Biochemical systems analysis: a study of function and design in molecular biology, volume 56. Addison-Wesley Reading, MA.

[10] Hofmeyr JHS, Cornish-Bowden A (1991) Quantitative assessment of regulation in metabolic systems. European Journal of Biochemistry 200: 223–236. 1879427 10.1111/j.1432-1033.1991.tb21071.x

[11] Fell D (1997) Understanding the control of metabolism. Portland Press Ltd.

[12] Koshland DE (1996) The structural basis of negative cooperativity: receptors and enzymes. Current Opinion in Structural Biology 6: 757–761. 8994875 10.1016/s0959-440x(96)80004-2

[13] Koshland DE, Hamadani K (2002) Proteomics and models for enzyme cooperativity. Journal of Biological Chemistry 277: 46841–46844. 12189158 10.1074/jbc.R200014200

[14] Cornish-Bowden A, Koshland D (1975) Diagnostic uses of the hill (logit and nernst) plots. Journal of molecular biology 95: 201–212. 171413 10.1016/0022-2836(75)90390-3

[15] Alvarado D, Klein DE, Lemmon MA (2010) Structural basis for negative cooperativity in growth factor binding to an EGF receptor. Cell 142: 568–579. 20723758 10.1016/j.cell.2010.07.015 PMC2925043

[16] Suzuki Y, Moriyoshi E, Tsuchiya D, Jingami H (2004) Negative cooperativity of glutamate binding in the dimeric metabotropic glutamate receptor subtype 1. Journal of Biological Chemistry 279: 35526. 15199056 10.1074/jbc.M404831200

[17] Pizard A, Marchetti J, Allegrini J, Alhenc-Gelas F, Rajerison RM (1998) Negative cooperativity in the human bradykinin b2receptor. Journal of Biological Chemistry 273: 1309. 9430662 10.1074/jbc.273.3.1309

[18] Bucher T, Russmann W (1964) Equilibrium and nonequilibrium in the glycolysis system. Ange-wandte Chemie International Edition in English 3: 426–439.

[19] Hofmeyr JHS, Cornish-Bowden A (1997) The reversible hill equation: how to incorporate coop-erative enzymes into metabolic models. Computer applications in the biosciences: CABIOS 13: 377–385. 9283752 10.1093/bioinformatics/13.4.377

[20] Gerhart JC, Pardee AB (1962) The enzymology of control by feedback inhibition. Journal of Biological Chemistry 237: 891–896. 13897943

[21] Levitzki A, Koshland DE (1969) Negative cooperativity in regulatory enzymes. Proceedings of the National Academy of Sciences 62: 1121. 10.1073/pnas.62.4.1121 PMC223623 5256410

