# Correction: Modeling the Role of Negative Cooperativity in Metabolic Regulation and Homeostasis

**DOI:** 10.1371/annotation/0e4bae85-55dd-4235-a39b-38d02cfd481e

**Published:** 2013-09-20

**Authors:** Eliot C. Bush, Anne E. Clark, Chris M. DeBoever, Lillian E. Haynes, Sidra Hussain, Singer Ma, Matthew B.A. McDermott, Adam M. Novak, John S. Wentworth

The name of the seventh author is incorrect. The correct name is: Matthew B.A. McDermott. 

